# Comparison of isolation platforms for detection of circulating renal cell carcinoma cells

**DOI:** 10.18632/oncotarget.21197

**Published:** 2017-09-23

**Authors:** Yvonne Maertens, Verena Humberg, Franziska Erlmeier, Sandra Steffens, Julie Steinestel, Martin Bögemann, Andres Jan Schrader, Christof Bernemann

**Affiliations:** ^1^ Clinic for Urology, University Hospital Muenster, Muenster, Germany; ^2^ Institute for Pathology and Pathological Anatomy, Technical University Munich, Munich, Germany

**Keywords:** clear cell renal cell carcinoma, circulating tumor cells, biomarker, liquid biopsy, genitourinary cancer

## Abstract

**Background:**

Analysis of circulating tumor cells (CTCs) has progressed in several tumor entities. However, little is known about CTCs in clear cell renal cell carcinoma (ccRCC) patients. Aim of our studies was to build a stable *in vitro* fundament for isolation of CTCs in ccRCC.

**Methods:**

We compared the analytical performance of different CTC isolation methods with regard to yield and purity: EpCAM based enrichment, leukocyte depletion and size based enrichment. EpCAM and cytokeratin 8 (KRT8) as biomarker for CTCs expression were evaluated in ccRCC cell lines as well as clinical samples.

**Results:**

While the EpCAM based approach failed to successfully isolate tumor cells, CD45 based approaches showed intermediate recovery rates. The cell-size based Parsortix system showed highest recovery rates. EpCAM expression was low or absent in most cell lines as well as in clinical samples, whereas KRT8 was detected as a potential biomarker in ccRCC.

**Conclusion:**

EpCAM based approaches might miss a high number of CTCs due to low or absent expression of EpCAM in ccRCC, as shown in cell lines as well as in patient samples. We identified the cell-sized based, label independent Parsortix system to be the most effective recovery system for ccRCC CTCs.

## INTRODUCTION

One of the hallmarks of cancer proposed by Hanahan and Weinberg is invasion and metastasis [1, 2]. Circulating tumor cells (CTCs), are probably key players within the metastatic cascade [1, 3, 4]. Over the past decade a plethora of studies have been published, describing the prognostic value of CTCs in different solid tumor entities [3–5]. Furthermore, CTC counts might also have the potential to serve as both predictive and prognostic real-time biomarker for the facilitation of treatment decisions [5–7].

So far, the only Food and Drug Administration (FDA) approved approach for CTC detection is the CellSearch system, which was first introduced in 2004 [6–9]. In this system, CTCs are enriched using a positive selection approach targeting EpCAM positive cells [8–10]. Thus, a CTC is defined being CD45-negative (a leukocyte marker) as well as positive for EpCAM, cytokeratin (CK) and 4′,6-diamidino-2-phenylindole (DAPI). However, other approaches, using negative selection or biophysical properties of CTCs are gaining more interest [10–13].

While several studies report detection and characterization of CTCs in tumor entities including breast or prostate cancer, the number of reports describing CTC detection in clear cell renal cell carcinoma (ccRCC) patients is limited. Most initial studies describe whole blood nucleic acid extraction and analysis [11–16] or CD45-negative selection [6, 14–16]. In a comparison of different tumor entities using the CellSearch system, ccRCC showed the lowest frequency of CTCs compared to all other tumor entities [6]. Another study detected CTCs as well as “suspicious objects” using the CellSearch system in metastatic RCC [17]. So far, however, there is no consistent classification for CTC determination in ccRCC patients. Thus, reliable and accurate methods for detection and analyses of CTCs are still missing.

**Figure 1:**
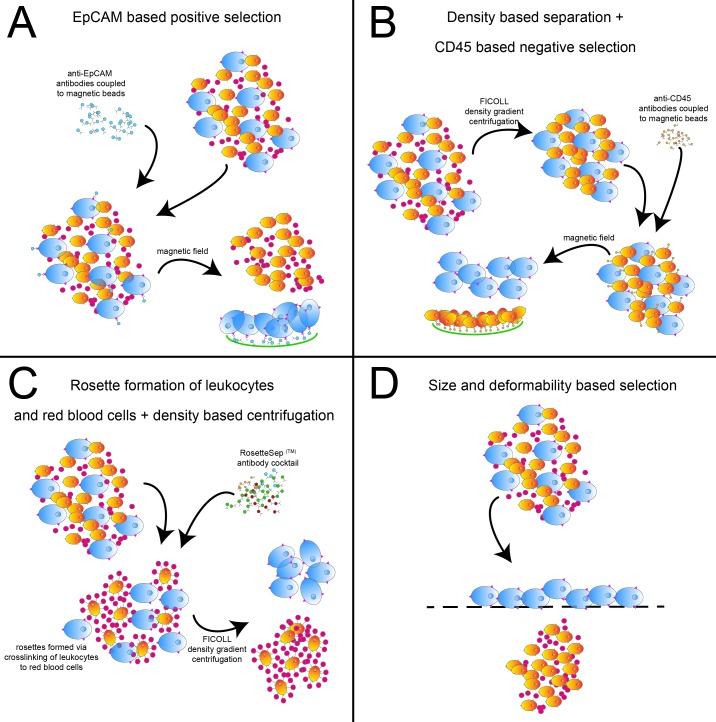
CTC isolation approaches Shown are the sequences of the 4 different CTC isolation approaches. **(A)** EpCAM-based positive enrichment using EpCAM beads. **(B)** Ficoll gradient centrifugation followed by negative enrichment using CD45 beads. **(C)** Negative enrichment with RosetteSep^™^ along with Ficoll gradient centrifugation. **(D)** Size and deformability based enrichment using the Parsortix system.

Here we performed comparative analyses of four different CTC enrichment strategies, based on either positive or negative selection approaches as well as biophysical properties of CTCs, i.e. size and deformability (Figure 1).

## RESULTS

### Leukocyte contamination

**Figure 2:**
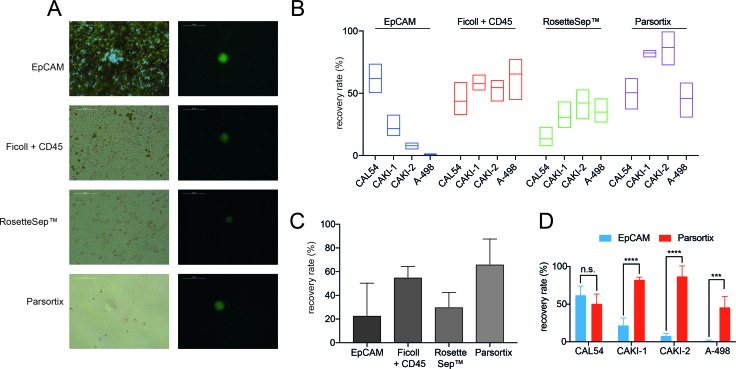
Analysis of purity and recovery rates of different CTC isolation approaches **(A)** Purity of different approaches. Shown are the images of isolation harvests to dissect the number of remaining leukocytes (brightfield, left). ccRCC tumor cells are shown in green (right). **(B)** Recovery rates of different CTC isolation approaches using 4 distinct ccRCC cell lines CAL-54, CAKI-1, CAKI-2 and A-498. **(C)** Median recovery rates of the different isolation approaches. **(D)** Comparison of recovery rates of EpCAM based and size based Parsortix system (n.s. not significant; ^***^ = p< 0.001; ^****^ = p< 0.0001).

When analyzing the purity of the recovery samples, we found few remaining leukocytes in the Parsortix harvest (Figure 2A). The highest contamination of leukocytes was found in the Ficoll/CD45 sample. Little contamination was detected in the RosetteSep^™^ system. The EpCAM system harvest contained very high numbers of magnetic beads bound to the tumor cells, making an estimation of contaminating leukocytes impossible.

### Recovery rates

Recovery rates of the EpCAM antibody immunomagnetic bead system were 61% for the CAL-54 cell line, 33% for CAKI-1 and only 0% – 10% for CAKI-2 and A498 (Figure 2B). Using the Ficoll density centrifugation followed by negative leukocyte depletion using CD45 magnetic beads we measured recovery rates between 32% (CAL-54) and 77% (A-498). The RosetteSep^™^ system showed recovery rates between 7% (CAL-54) and 53% (CAKI-2). The highest recovery rates ranging from 30% (A-498) up to 87% (CAKI-2) were detected using the Parsortix system. When calculating the median recovery rate, the Parsortix system showed the highest median rate with 66%, followed by Ficoll/CD45 with 55%, RosetteSep with 30% and EpCAM with 23% median recovery rate (Figure 2C). These results demonstrate a low efficacy of CTC isolation in ccRCC cell lines using an EpCAM based approach.

Since the only FDA approved system for CTC detection is based on EpCAM expression we performed deeper comparison of the recovery rates of the EpCAM based system compared to the size and deformability based Parsortix system. We detected almost similar recovery rates in the ccRCC cell line CAL-54 using both the EpCAM based system and the Parsortix system with median recovery rates of 61% and 51%, respectively (Figure 2D). In all three remaining cell lines, however, the Parsortix system achieved significantly higher recovery rates (CAKI-1: 82% vs. 21% (p< 0.0001); CAKI-2: 87% vs. 7% (p< 0.0001), and A-498: 46% vs. 1% (p< 0.001)).

### EpCAM and cytokeration expression in ccRCC

**Figure 3:**
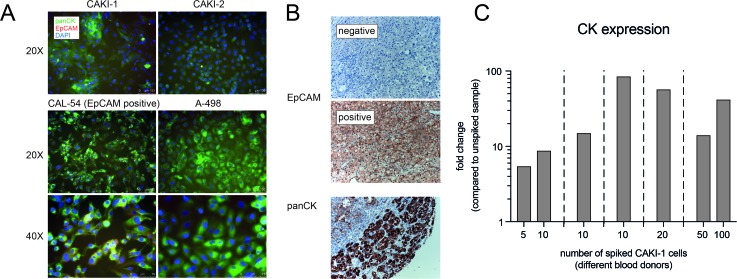
Analysis of potential CTC biomarkers in cell lines and RCC tissue samples **(A)** Immunofluorescence analysis of panCK and EpCAM expression in 4 distinct ccRCC cell lines. **(B)** Immunohistochemical analysis of EpCAM and panCK expression in clinical ccRCC tissue samples. **(C)** qPCR analysis of distinct spiking experiments showing induction of KRT8 expression.

Subsequently, we analyzed the expression of established 'CellSearch CTC definition markers' EpCAM and cytokeratins in both ccRCC cell lines as well as clinical samples. We performed immunofluorescence analysis on cell lines used for spiking experiments. We detected expression of panCK in all cell lines, whereas EpCAM was exclusively expressed weakly in CAL-54 cells (Figure 3A). Furthermore, we performed immunohistochemistry for panCK and EpCAM on 61 ccRCC tissue samples. All tumors showed a diffuse positive staining for panCK; 18 of 61 cases (29%) showed EpCAM expression (Figure 3B). There was no correlation to a certain tumor grading, as percentage of EpCAM positive samples ranged between 23% in grade 3 RCC, 24% in grade 1, and 34% in grade 2 RCC, respectively.

### Cytokeratin KRT8 as a potential marker of ccRCC CTCs

Finally, we performed spiking experiments using different numbers of CAKI-1 cells spiked into healthy blood samples, followed by Parsortix isolation and TaqMan qPCR for detection of CK expression. We could continuously detect robust induction of CK expression in spiked samples compared to unspiked controls (Figure 3C).

## DISCUSSION

Analysis of “liquid biopsy” components, i.e. CTCs, circulating cell-free DNA or exosomes, can offer improvement in cancer therapies. However, analytical and clinical validity have to be sufficient to meet FDA standardized criteria for use in clinical settings [6, 18]. Hence, in ccRCC, CTCs have not been analyzed in standardized settings, making the application as a clinical biomarker difficult in its present form. Therefore, we aimed to analyze the most effective strategy for isolation of CTCs from patients with ccRCC.

Recovery rate comparison of ccRCC tumor cell lines revealed the lowest median recovery using the EpCAM based enrichment approach in three out of four ccRCC cell lines. Only one cell line (CAL-54) exhibited a high recovery rate of 61%. Accordingly, immunofluorescence analysis of the 4 tested ccRCC cell lines revealed EpCAM expression only in CAL-54 cell with no significant EpCAM expression in the other three cell lines used in our study. By performing immunohistochemistry analysis of primary RCC tissue, EpCAM expression was detected in only 29% of the tumor samples, which is in line with previous reports showing low expression of EpCAM in ccRCC tissues [19, 20]. This demonstrates that an EpCAM based CTC enrichment strategy is not appropriate for extensive detection of CTCs in patients with ccRCC. This is in accordance with several study results demonstrating a very small number of detectable CTCs in RCC patients compared to other solid tumors when using the CellSearch system [6, 14, 16].

Besides positive enrichment of CTCs, alternative approaches have been developed to deplete non-malignant cells from the blood sample, especially leukocytes. The usage of a density based isolation protocol of PBMCs in combination with immunomagnetic depletion of CD45+ cells has been described to successfully enrich for CTCs [14, 16, 21, 22]. In our study, density centrifugation followed by CD45 depletion showed comparable recovery rates to the EpCAM based enrichment strategy – at least when analyzing EpCAM positive cells, e.g. CAL-54. Additionally, it greatly improves the recovery rate of ccRCC cells without EpCAM expression. However, this procedure results in a high number of contaminating leukocytes. This contamination needs to be considered in downstream applications, e.g. biomarker analyses by qPCR. The RosetteSep^™^ system, which is also based on depletion of blood cells, showed a pure sample recovery, with low leukocyte contamination. However, the highest recovery rate of this system was only about 40%. Summing up, both leukocyte depletion assays, Ficoll/CD45 as well as the RosetteSep^™^ system, displayed weaknesses regarding either purity or efficiency, making clinical usage less promising.

The highest median recovery rate was achieved by using the Parsortix system, a label independent enrichment platform. This system showed a highest median recovery rate of 87% in CAKI-2 cells. Furthermore, this technique resulted in pure recovery with lowest number of contaminating leukocytes. This system has already been used for detection of CTCs from different tumor entities, e.g. breast or prostate cancer [21–23]. In small cell lung cancer the Parsortix system showed comparable efficiency to the CellSearch system for enrichment of EpCAM positive cells, but greatly improved recovery of EpCAM negative or EpCAM low expressing cells [23]. Chudziak et al. concluded that the Parsortix system enables the detection of an additional subset of CTCs which might otherwise be missed when using epitope dependent systems i.e. EpCAM. This is in line with our results demonstrating a significant improvement of recovery by using the Parsortix system over the EpCAM based enrichment strategy. However, other enrichment approaches which are not based on EpCAM expression might also offer strategies for CTC isolation in ccRCC patients. Accordingly, Liu et al. recently described comparable capture efficiency using a combination of surface proteins, i.e. Carboanhydrase 9 (CA9) and CD147 [24].

CK expression is a major characteristic of CTCs [7]. We observed ubiquitous expression of CK in RCC tumor samples as well as in all cell lines. By performing spiking experiments with different numbers of cells, we could consecutively detect expression changes of KRT8 mRNA, making KRT8 and other members of the CK family potential markers for detection of ccRCC CTCs.

By performing a comprehensive comparison of 4 different CTC enrichment strategies we found the Parsortix system displaying the highest recovery rate and the lowest leukocyte contamination. Therefore, the EpCAM independent Parsortix system shows the highest potential for successful ccRCC CTC enrichment. This is in line with the observation of reduced EpCAM expression in ccRCC. Thus, use of the label independent Parsortix system in combination with biomarker analysis, i.e. expression of CK, might improve the therapeutic monitoring of ccRCC therapies.

The use of different established ccRCC cell lines enabled us to illustrate distinct immunological and phenotypical profiles, conferrable to the phenotypic heterogeneity among clinical ccRCC samples. However, the main limitation of our study is the absence of blood samples from ccRCC patients. Further analyses using clinical blood samples in combination with non-EpCAM based approaches are necessary and will gain insights into the biology of CTCs and presumably the use of liquid biopsy in patients with ccRCC.

## MATERIALS AND METHODS

### Cell culture

ccRCC cell lines CAKI-1, CAKI-2, CAL-54 and A-498 cells were purchased from Leibnitz Institute DSMZ – German Collection of Microorganism and Cell Cultures (Braunschweig, Germany). Cells were thawed, expanded and cultured under appropriate conditions. Cells were used only from passage 7 to 20. For fluorescence labeling cell lines were labelled with a green fluorescent dye (CellTracker^™^ Green CMFDA Dye, Thermo Fisher Scientific, Waltham, MA, USA).

### Enrichment approaches

1. EpCAM-based positive enrichment using Epithelial Enrich Dynabeads (Invitrogen, Thermo Fisher Scientific, Waltham, MA, USA): 100 μl beads per 5 ml blood were used for magnetic enrichment of EpCAM positive cells according to manual (Figure 1A).

2. Gradient centrifugation with Ficoll-Paque and negative enrichment using CD45-Dynabeads (Invitrogen, Thermo Fisher Scientific, Waltham, MA, USA): Blood samples were diluted with the double amount of isolation buffer (Dulbecco's PBS w/o Ca^2+^ und Mg^2+^, with 0,1 % BSA and 2 mM EDTA, all PAA Laboratories, GE Healthcare, Chicago, IL, USA) and layered over 15 ml Ficoll-Paque PLUS (GE Healthcare, Chicago, IL, USA). After gradient centrifugation (40 min at room temperature and 500 x g), PBMCs were harvested from the interphase. The harvest was washed twice in an appropriate volume of isolation buffer. Finally, the harvest was resuspended in 1 ml isolation buffer. 150 μl Dynabeads per 5 ml blood were resuspended with the PBMC suspension and incubated 30 min at 4 °C with gentle tilting and rotation. After incubation, beads and bound cells were depleted into a magnetic field while supernatant with unbound cells was transferred to a new tube (Figure 1B).

3. Gradient centrifugation with Ficoll-Paque and negative enrichment with RosetteSep^™^: 250 μl RosetteSep^™^ Human CD45 Depletion Cocktail (STEMCELL Technologies, Vancouver, Canada) was directly added to the blood sample and mixed. The sample was incubated 20 min at room temperature, mixing again after 10 min. After incubation, the mixture was diluted with the double amount of isolation buffer (Dulbecco's PBS w/o Ca^2+^ and Mg^2+^, with 2 % FBS, all PAA Laboratories, GE Healthcare, Chicago, IL, USA) and layered over 15 ml Ficoll-Paque PLUS. After gradient centrifugation (20 min at room temperature and 1200 x g) cells were harvested from the interphase. The harvest was washed twice in an appropriate volume of isolation buffer (Figure 1C).

4. Enrichment using Parsortix (ANGLE plc, Guildford, UK) system: The blood sample was processed using the 6.5 μm separation cassettes according to the manufacturer's protocol. Briefly, the separation cassette was prepared for isolation by running the priming protocol. After priming of the separation cassette, the blood sample (5ml) was loaded onto the device and the separation procedure was started. After separation and intermediate cleaning of the device, the captured cells were harvested from the cassette by inverting the flow direction. The harvest was flushed from the cassette in 200 μl PBS (Figure 1D).

### Recovery rate determination

Fluorescence labeled cells were harvested with Trypsin/EDTA (PAA) and 200 cells were sorted using flow cytometry (BD FACSAria^™^, BD Biosciences, Franklin Lakes NJ, USA). Tumor cells were spiked into blood samples (5 ml) from healthy blood donors. The spiked samples were processed using the four different isolation technologies (Figure 1). After enrichment, collected cells were transferred to a 96-well microplate and counted manually by fluorescence microscopy. All experiments were performed in three biological replicates. Statistical analysis was performed via Sidak's multiple comparison tests using GraphPad Prism 7 (GraphPad Software, Inc, La Jolla, CA, USA).

### Immunocytochemistry

For immunocytochemistry, cells were fixed and permeabilized using 4% paraformaldehyde / 0.1% Triton X-100 in PBS, and blocked with 5% FCS / 2% BSA / 2% glycine in PBS-T (all Sigma Aldrich, St. Louis, MO, USA). Primary antibodies were applied in 0.5% BSA / 0.5% glycine / PBS-T overnight at 4°C: pan-Keratin (C11) conjugated with Alexa Fluor 488 (1:150, Cell Signaling, Danvers, MA, USA) and EpCAM (VU-1D9) conjugated with PerCP/Cy5.5 (1:200, Abcam, Cambridge, MA, USA) along with DAPI (4′,6-diamidino-2-phenylindole, 1:400, Sigma Aldrich, St. Louis, MO, USA).

### Immunohistochemistry (IHC)

Sixty-one patients, who had undergone renal surgery at the department of urology of the Technical University Munich for ccRCC, were identified using the electronic pathology register. For each tissue sample, relevant clinico-pathological attributes were available. One pathologist (FE) selected suitable specimens, and tissue micro arrays (TMA) were prepared from the primary tumor blocks as previously described [25]. The collective included 15, 29, and 17 ccRCC samples from patients with grade 1, 2, and 3 carcinomas, respectively. Expression of CKpan and EpCAM was determined by IHC. The 2 μm formalin-fixated and paraffin-embedded TMA-slides were stained for CKpan and EpCAM in a fully automated Benchmark XT immunostainer (Ventana Medical Systems, Tucson, AZ, USA). Antigen retrieval was accomplished at pH 8.4. CKpan and EpCAM expression was detected by commercially available antibodies (CKpan MNF 116, #CI62IR06, DCS; Anti-EpCAM, #71916, Abcam, Cambridge, MA, USA). The optimal dilution for CKpan was: 1:200 and for EpCAM 1:100. For visualization of bound primary antibody, the ultraView Universal DAB Detection Kit (Ventana Medical Systems, Tucson, AZ, USA) was used. Afterwards, sections were briefly rinsed in tap water, counterstained with Mayer's Hematoxylin solution and then mounted. All stained tissue samples were assessed in a blind study by a pathologist (FE). The evaluation was performed under a Leitz ARISTOPLAN light microscope (Leica Microsystems, Wetzlar, Germany) with a x10 eyepiece, a 22-mm field of view and x40 objective lens (Plan FLUOTAR x40/0.70). The study was carried out according to the latest version of the Declaration of Helsinki and approved by the institutional ethics committee (412/16S).

### Quantitative real time PCR (qPCR)

For qPCR analysis, harvested cells were lysed by adding 200 μl 2X lysis buffer (200 mM Tris-HCl, 1 μM LiCl, 20 mM EDTA, 2 % LiDS and 10 mM DTT, all Sigma Aldrich, St. Louis, MO, USA). Mature mRNA was isolated from the lysed samples with the Dynabeads mRNA DIRECT^™^ Kit (Invitrogen, Thermo Fisher Scientific, Waltham, MA, USA) according to manual. Immediately, isolated mRNA was transcribed to cDNA using the Sensiscript RT Kit (Qiagen, Hilden, Germany) according to manual. RNA degradation during reverse transcription was prevented by adding 0,25 μl RNasin RNase PLUS Inhibitor (Promega, Madison, WI, USA). qPCR was performed using TaqMan Fast Advanced Master Mix (Applied Biosystems) and TaqMan-Assays for KRT8 (Hs01595539_g1), RPL37A (Hs01102345_m1) and HPRT1 (Hs99999909_m1) (all Thermo Fisher Scientific, Waltham, MA, USA). qPCR was performed using a QuantStudio 3 cycler. QuantStudio Design & Analysis Sofware v1.1 (Applied Biosystems, Thermo Fisher Scientific, Waltham, MA, USA) was used for analysis.

## Abbreviations

CTC: circulating tumor cells
ccRCC: clear cell renal cell carcinoma
cDNA: complimentary DNA
CK: cytokeratin
DAPI: 4′,6-diamidino-2-phenylindole
min: minutes
ml: milliliter
mM: millimolar
mRNA: messenger RNA
qPCR: quantitative polymerase chain reaction
RCC: renal cell carcinoma
RT: Reverse Transcription
TMA: tissue microarray
μl: microliter
μm: micrometer
PBMCs: peripheral blood mononuclear cells
